# Effects of a national quality improvement program on ICUs in China: a controlled pre-post cohort study in 586 hospitals

**DOI:** 10.1186/s13054-020-2790-1

**Published:** 2020-03-04

**Authors:** Huaiwu He, Xudong Ma, Longxiang Su, Lu Wang, Yanhong Guo, Guangliang Shan, Hui Jing He, Xiang Zhou, Dawei Liu, Yun Long, Yupei Zhao, Shuyang Zhang, China-NCCQC group

**Affiliations:** 1 Department of Critical Care Medicine, Peking Union Medical College Hospital, Peking Union Medical College and Chinese Academy of Medical Sciences, Beijing, 100730 China; 2 Department of Medical Administration, National Health Commission of the People’s Republic of China, Beijing, 100044 China; 3 Department of Epidemiology and Biostatistics, Institute of Basic Medicine Sciences, Chinese Academy of Medical Sciences (CAMS) & School of Basic Medicine, Peking Union Medical College, Beijing, 100730 China; 4 Department of General Surgery, Peking Union Medical College Hospital, Peking Union Medical College and Chinese Academy of Medical Sciences, Beijing, 100730 China; 5 Department of Cardiology, Peking Union Medical College Hospital, Peking Union Medical College and Chinese Academy of Medical Sciences, Beijing, 100730 China

**Keywords:** Medical quality, ICU, Quality improvement (QI) program, China

## Abstract

**Introduction:**

Patient safety and critical care quality remain a challenging issue in the ICU. However, the effects of the national quality improvement (QI) program remain unknown in China.

**Methods:**

A national ICU QI program was implemented in a controlled cohort of 586 hospitals from 2016 to 2018. The effects of the QI program on critical care quality were comprehensively investigated.

**Main results:**

A total of 81,461,554 patients were enrolled in 586 hospitals, and 1,587,724 patients were admitted to the ICU over 3 years. In 2018, there was a significantly higher number of ICU beds (2016 vs. 2018: 10668 vs. 13,661, *P* = 0.0132) but a lower doctor-to-bed ratio (2016 vs. 2018: 0.64 (0.50, 0.83) vs. 0.60 (0.45, 0.75), *P* = 0.0016) and nurse-to-bed ratio (2016 vs. 2018: 2.00 (1.64, 2.50) vs. 2.00 (1.50, 2.40), *P* = 0.031) than in 2016. Continuous and significant improvements in the ventilator-associated pneumonia (VAP) incidence rate, microbiology detection rate before antibiotic use and deep vein thrombosis (DVT) prophylaxis rate were associated with the implementation of the QI program (VAP incidence rate (per 1000 ventilator-days), 2016 vs. 2017 vs. 2018: 11.06 (4.23, 22.70) vs. 10.20 (4.25, 23.94) vs. 8.05 (3.13, 17.37), *P* = 0.0002; microbiology detection rate before antibiotic use (%), 2016 vs. 2017 vs. 2018: 83.91 (49.75, 97.87) vs. 84.14 (60.46, 97.24) vs. 90.00 (69.62, 100), *P* < 0.0001; DVT prophylaxis rate, 2016 vs. 2017 vs. 2018: 74.19 (33.47, 96.16) vs. 71.70 (38.05, 96.28) vs. 83.27 (47.36, 97.77), *P* = 0.0093). Moreover, the 6-h SSC bundle compliance rates in 2018 were significantly higher than those in 2016 (6-h SSC bundle compliance rate, 2016 vs. 2018: 64.93 (33.55, 93.06) vs. 76.19 (46.88, 96.67)). A significant change trend was not found in the ICU mortality rate from 2016 to 2018 (ICU mortality rate (%), 2016 vs. 2017 vs. 2018: 8.49 (4.42, 14.82) vs. 8.95 (4.89, 15.70) vs. 9.05 (5.12, 15.80), *P* = 0.1075).

**Conclusions:**

The relationship between medical human resources and ICU overexpansion was mismatched during the past 3 years. The implementation of a national QI program improved ICU performance but did not reduce ICU mortality.

## Key messages

- To the best of our knowledge, the present study has the largest sample among investigations of quality control in ICUs in the world. The present study reflected the national policy on ICU quality control and the impact factor in China.
- The present study reflected the development of the ICU in China. Interestingly, we found that the human resources, that is, the number of doctors and nurses, might not have kept pace with the expansion of the ICUs in China over the past 3 years. An imbalanced relationship between ICU expansion and human resources was found in this study. The health administration department should pay attention to ICU overextension, which might negatively affect quality.
- The national QI program was validated in the present study; the program resulted in significant improvements in the VAP incidence rate, microbiology detection rate before antibiotic use, DVT prophylaxis rate, and the 3-h and 6-h SSC bundle compliance rates.

## Introduction

In recent decades, critical care medicine has developed rapidly worldwide [1, 2]. The irreplaceable role of rescue treatment for critically ill patients has been widely accepted in intensive care units (ICUs). However, the severity of critically ill patients, a high intensity of therapeutic activities per day, a stressful environment, the quantity and complexity of medical data, and sudden changes in the patient’s condition cause ICUs to be associated with high risks of medical errors, hospital-acquired infections, and medical complications. Studies have reported that there is a high rate of medical errors and adverse event occurrence in ICUs [3]. Valentin et al. found that sentinel events related to medication, indwelling lines, airway clearance, and equipment failure in ICUs occur with considerable frequency [4]. Furthermore, an increasing number of evidence-based practices associated with acquired infection prevention and sepsis resuscitation might improve critically ill patient outcomes in the ICU, but eligible patients may not receive them. Hence, there is an urgent need for the development and implementation of new strategies with the aim of improving care quality in ICUs.

The effect of quality improvement (QI) programs on clinical practice and outcomes is gaining increasing attention in critical care medicine. Garrouste-Orgeas et al. reported that a multifaceted program was effective at preventing insulin errors and accidental tube/catheter removal [5]. Moreover, Cavalcanti et al. found that the implementation of a multifaceted QI intervention with daily checklists, goal setting, and clinician prompting did not reduce in-hospital mortality among critically ill patients treated in ICUs in Brazil [6]. A cluster randomized trial showed that multifaceted QI interventions improved the adoption of care practices in community ICUs [7]. Recently, McCredie et al. found that a trauma QI program improved clinical outcomes in severe traumatic brain injury patients [8].

However, the effect of a national QI program on ICU performance remains unknown in China. We sought to investigate the effects of a national QI program on ICUs in China. We assessed the association between this intervention and critical care quality performance and ICU mortality. Moreover, potential factors such as regional economic income and hospital/ICU structure resources were considered in the assessment of the effect of the interventions.

## Methods

The study protocol was approved by the institutional review board of Peking Union Medical College Hospital; the approval included a waiver for the informed consent of the patients and physicians.

We used a pre-post cohort design to evaluate the implementation of a national QI program in ICUs in China. This program was led by the China National Critical Care Quality Control Center (China-NCCQC), which is the official national department that regulates ICU quality control in China. This program was considered an administrative strategy in these pilot hospitals.

### Intervention

A multifaceted QI program was implemented in the involved hospitals. The intervention comprised three components with multiple elements:

1. . Construction of the ICU quality control team: the involved hospitals were required to build ICU quality control teams with the aim of improving ICU performance. Each ICU designated one physician and one nurse as the team leaders. The quality control team was responsible for auditing ICU quality performance and collecting and analyzing the ICU quality indicators.
2. . Training: the local team special contractor received information on how to assess the training regarding how to improve ICU quality performance and then disseminated this information among their colleagues in the ICU. The related materials focused on the bundles for the prevention of ventilator-associated pneumonia (VAP), catheter-related bloodstream infection (CRBSI), deep vein thrombosis (DVT), catheter-associated urinary tract infection (CAUTI), and sepsis resuscitation.
3. . Performance of the provincial and national audits and feedback: the related data of the ICU quality control indicators were submitted online to the China-NCCQC annually. The provincial and national educational sessions were held annually and were attended by all ICU staff members. Interventions included in the training program are shown in Additional file 1.

### Study population and setting

The study was divided into two phases. In the first phase, the quality control team was constructed, and staff were trained regarding data recording and reporting; this phase occurred in 2016. The quality data were collected annually. The data for the entire year of 2016 were taken as the baseline. In the second phase, the QI program was continuously implemented. The China-NCCQC collected the relevant data regarding the quality control indicators through the database of the National Clinical Improvement System (https://ncisdc.medidata.cn/login.jsp). The enrolled hospitals (each included ICU had to have more than five beds) voluntarily participated and were selected by the China-NCCQC. The selection criteria of the China-NCCQC were as follows: (1) The ICU has the ability to diagnose and treat the relevant medical quality control items (such as DVT, VAP, and CRBSI). (2) The enrolled ICUs met the requirements for equipment, construction, and management of ICUs in China [9–11].

### Study measurements

A trained data collector in each ICU was required to submit and report the quality control data via the internet. The special investigators (S.L.Y. and L.W.) from the China-NCCQC handled the data submitted from each ICU. The 15 related ICU quality control indicators have been broadly discussed, and a consensus was reached by ICU experts in 2013 in China. Moreover, these indicators were officially recommended for the assessment of ICU performance by the National Health Commission of the People’s Republic of China on 10 April 2015. Among the 15 quality control indicators, there are 3 structural indicators (proportion of ICU patients out of all total inpatients, proportion of ICU bed occupancy out of the total inpatient bed occupancy, proportion of ICU patients with acute physiology, and chronic health evaluation (APACHE) II scores ≥ 15 out of all ICU patients), 4 process indicators (3-h Surviving Sepsis Campaign (SSC) bundle compliance rate, 6-h SSC bundle compliance rate, microbiology detection rate before antibiotic use, DVT prophylaxis rate), and 8 outcome indicators (unplanned endotracheal extubation rate, reintubation rate within 48 h, rate of unplanned transfer to ICU, ICU readmission rate within 48 h, VAP incidence rate, CRBSI incidence rate, CAUTI incidence rate, ICU mortality). The definitions and meanings of the 15 quality control indicators are described in Table 1. Moreover, the quality control protocol for the collected data is shown in Additional file 2.

**Table 1:** National clinical quality control indicators for critical care

Indicators	Definition and meaning
Proportion of ICU patients to total inpatients (%)	**Definition**: (patients admitted to the ICU)/(patients admitted to hospital during the same period) **Meaning**: reflects the proportion and hospital course of ICU patients among all hospitalized patients
Proportion of ICU patient bed occupancy to total inpatient bed occupancy (%)	**Definition**: (days of ICU bed occupancy by patients)/(days of hospital bed occupancy by patients during the same period) **Meaning**: reflects the proportion and hospital course of ICU patients among all hospitalized patients
Proportion of ICU patients with APACHE II score ≥ 15 (%) (within 24 h after being admitted to ICU)	**Definition**: (no. of patients with APACHE II score ≥ 15 during the first 24 h in the ICU)/(patients admitted to the ICU during the same period) **Meaning**: reflects the severity of illness of patients admitted to ICU.
3-h SSC bundle compliance rate (%)	**Definition**: (no. of septic shock patients who received the 3-h SSC bundle treatment)/(no. of septic shock patients admitted to the ICU during the same period) **Meaning**: reflects the clinical standardization and medical care capacity for septic shock
6-h SSC bundle compliance rate (%)	**Definition**: (no. of septic shock patients who received the 6-h SSC bundle treatment)/(no. of septic shock patients admitted to the ICU during the same period) **Meaning**: reflects the clinical standardization and medical care capacity for septic shock.
Microbiology detection rate before antibiotic use (%)	**Definition**: (no. of patients with microbiology detection before antibiotics)/(no. of patients who received antibiotics during the same period) **Meaning**: reflects the normative use of antibiotics in ICU.
DVT prophylaxis rate (%)	**Definition**: (no. of patients who received DVT prophylaxis treatment)/(no. of patients admitted to the ICU during the same period). The optimal mode of DVT prophylaxis includes pharmacological prophylaxis (heparin or LMWH), mechanical prophylaxis (intermittent pneumatic leg compression or elastic stockings), and inferior vena cava filter. **Meaning**: assesses the DVT prophylaxis for ICU patients.
Unplanned endotracheal extubation rate (%)	**Definition**: (no. of patients with unplanned endotracheal extubation)/(no. of patients with endotracheal extubation during the same period) **Meaning**: reflects the integral management and treatment level of ICU.
Reintubation rate within 48 h (%)	**Definition**: (no. of patients reintubated within 48 h after endotracheal extubation)/(no. of patients with endotracheal extubation during the same period) **Meaning**: reflects the judgment of proper extubation indication in ICU.
Rate of unplanned transfer to ICU (%)	**Definition**: (no. of patients with unplanned transfer to the ICU from other wards)/(no. of patients transferred to the ICU from other wards during the same period) **Meaning**: reflects the quality of medical care in medical institutions.
ICU readmission rate within 48 h (%)	**Definition**: (no. of patients readmitted to the ICU within 48 h after discharge from the ICU)/(no. of patients discharged from the ICU during the same period) **Meaning**: reflects the judgment of proper discharge indication in the ICU.
VAP incidence rate (%) per 1000 ventilator days	**Definition**: (no. of patients with VAP)/(no. of patients with mechanical ventilation during the same period) **Meaning**: reflects the capability of infection control, invasive mechanical ventilation, and disease management in the ICU.
CRBSI incidence rate (%) per 1000 catheter days	**Definition**: (no. of patients with CRBSI)/(no. of patients with a central venous catheter during the same period) **Meaning**: reflects the capability of infection control, intravascular catheter indwelling, and disease management in the ICU.
CAUTI incidence rate (%) per 1000 catheter days	**Definition**: (no. of patients with CAUTI)/(no. of patients with a urinary catheter during the same period) **Meaning**: reflects the capability of infection control, urinary catheter indwelling, and disease management in the ICU
ICU mortality rate (%)*	**Definition**: (no. of patients who died in the ICU)/(no. of patients admitted to the ICU during the same period)

* The ICU mortality rate was included in the present study. The standardized mortality ratio and expected mortality rate were also included in the original document of quality control indicators for critical care medicine, released by the National Health Commission of the People’s Republic of China at 2015

### Statistical analysis

All statistical analyses were performed in SAS 9.4 (SAS Institute Inc., Cary, NC, USA). The study design involved clustering by hospital. All provinces were equally divided into low-, middle-, and high-income groups based on the 2016 real gross domestic product (GDP) per capita of each province from the 2016 China Statistical Yearbook.

We compared the parameters (hospitals and ICU organizational structure and ICU quality control indicators) by hospital level (secondary and tertiary), year (2016, 2017, and 2018), and GDP (low, middle, and high) using the Wilcoxon rank-sum test (2-sample). Spearman’s rank correlation coefficient was used to test the changing trend of the parameters from 2016 to 2018. Data are presented as median values (25th–75th interquartile ranges [IQRs]), numbers (%), or odds ratios (ORs) (95% confidence intervals [CIs]) as appropriate. All statistical tests were two-tailed, and a *P* value less than 0.05 was considered statistically significant.

## Results

### Characteristics of the total study group

**Fig. 1:**
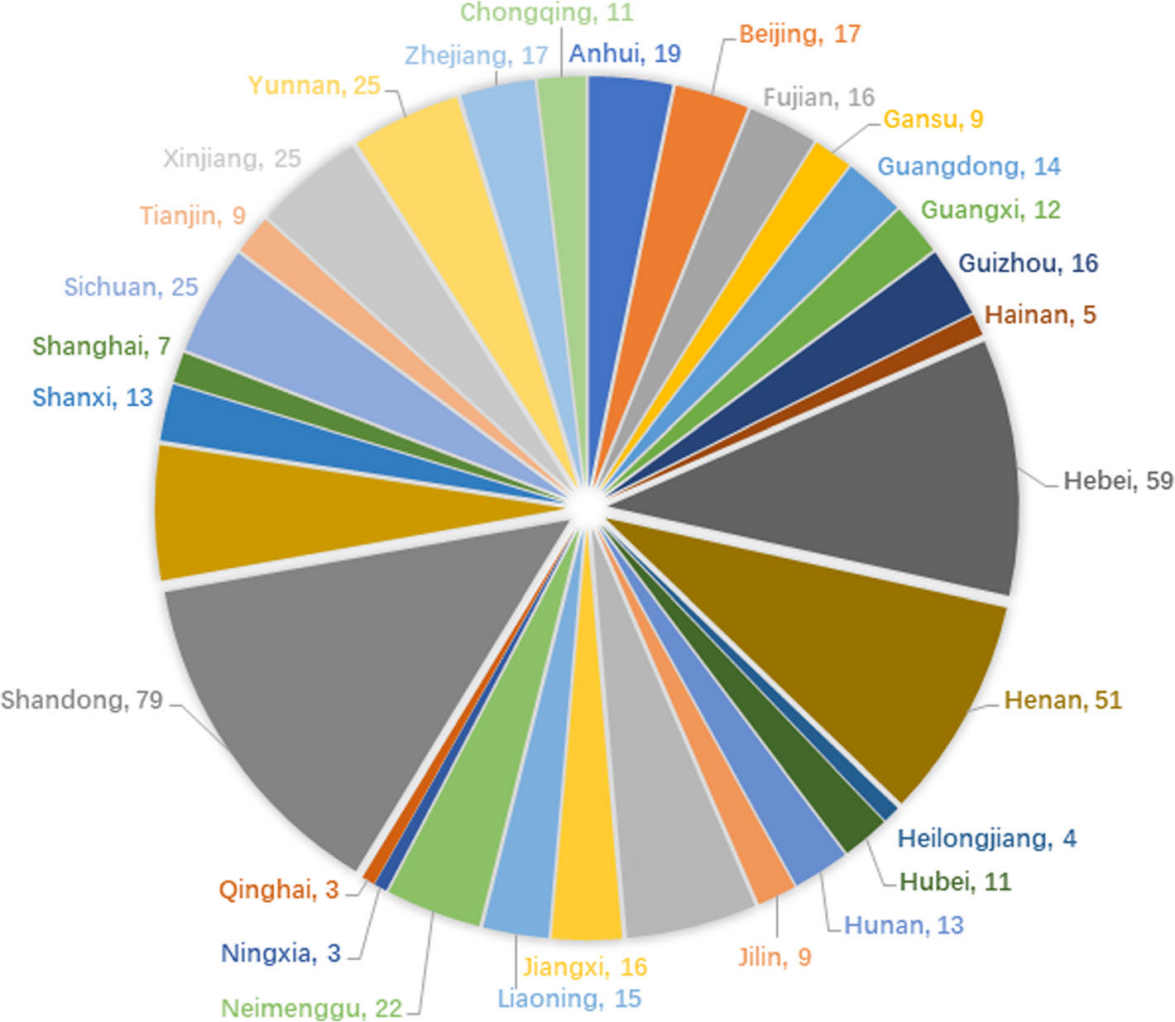
The numbers of hospitals in 30 provinces of China

A total of 586 hospitals from 30 provinces were enrolled in the ICU QI program from 2016 to 2018. The proportions of hospitals from each province are shown in Fig. 1. Most provinces of mainland China joined this program except Tibet. A total of 81,461,554 patients were admitted to the hospitals, and 1,587,724 patients were admitted to the ICUs during the 3 years.

### Hospital and ICU organizational characteristics

All hospital and ICU organizational characteristics were analyzed and are presented in Table 2. In 2018, there was a significantly higher number of ICU beds (2016 vs. 2018: 10668 vs. 13,661, *P* = 0.0132) but lower doctor-to-bed (2016 vs. 2018: 0.64 (0.50, 0.83) vs. 0.60 (0.45, 0.75), *P* = 0.0016) and nurse-to-bed (2016 vs. 2018: 2.00 (1.64, 2.50) vs. 2.00 (1.50, 2.40), *P* = 0.031) ratios compared with the values in 2016. Moreover, a significant continuously increasing trend in the total number of admitted patients in hospital and ICU, the number of ICU beds, but a decreasing trend of doctor-to-bed ratio in ICU were found from 2016 to 2018 (shown in Additional file 3).

**Table 2:** Hospitals and ICU organizational characteristics

Parameters	2016	2017	2018	Trend *P* value
Total no. of admitted patients	26,282,732	27,321,537	27,857,285	
Median (25th–75th)	31,973(20367–51,045)	34,864(21881–57,119)	37,252(23034–59,531)	0.0013
Total no. of admitted patients in ICU	471,432	526,663	589,629	
Median(25th–75th)	519(297–851)	562(334–927)	610(351–1055)	0.0002
Total no. of hospital beds	612,441	680,429	691,511	
Median(25th–75th)	600(941–1446)	997(605–1500)	993(650–1552)	0.0580
Total no. of ICU beds	10,668	11,355	13661*	
Median(25th–75th)	12(9–20)	13(10–20)	15(10–24)	0.0002
ICU: hospital bed percentage(%)	1.5(1.1–2)	1.5(1.1–2)	1.6(1.2–2.1)*†	0.0018
Doctor-to-nurse ratio in ICU	0.31 (0.26, 0.38)	0.31 (0.25, 0.38)	0.30 (0.25, 0.38)	0.1949
Doctor-to-bed ratio in ICU	0.64 (0.50, 0.83)	0.60 (0.50, 0.80)	0.60 (0.45, 0.75)*	0.0005
Nurse-to-bed ratio in ICU	2.00(1.64, 2.50)	2.06 (1.63, 2.50)	2.00 (1.50, 2.40)*	0.0113

*vs.2016, *P* < 0.05;

^**†**^vs.2017, *P* < 0.05;

### Effects of the QI program on ICU performance

The changes in the 15 preset quality indicators from 2016 to 2018 are shown in Table 3. Significant continuous improvements in the VAP incidence rate, microbiology detection rate before antibiotic use, and DVT prophylaxis rate were found during the implementation of the QI program (VAP rate, 2016 vs. 2018 vs. 2018: 11.06 (4.23, 22.70) vs. 10.20 (4.25, 23.94) vs. 8.05 (3.13, 17.37), *P* = 0.0002; microbiology detection rate before antibiotic use, 2016 vs. 2018 vs. 2018: 83.91 (49.75, 97.87) vs. 84.14 (60.46, 97.24) vs. 90.00 (69.62, 100), *P* < 0.0001; DVT prophylaxis rate, 2016 vs. 2018 vs. 2018: 74.19 (33.47, 96.16) vs. 71.70 (38.05, 96.28) vs. 83.27 (47.36, 97.77), *P* = 0.0093). However, there was no significant change in ICU mortality rate during the implementation of the QI program from 2016 to 2018 (8.49% vs. 8.95% vs. 9.05%, *P* = 0.1075) (shown in Additional file 4).

**Table 3:** Change of 15 ICU quality control indicators from 2016 to 2018

Indicators	2016	2017	2018	Trend *P* value
Proportion of ICU in total inpatients(%)	1.60(1.13, 2.44)	1.63(1.13, 2.45)	1.63(1.12, 2.42)	0.7562
Proportion of ICU in total inpatient bed occupancy(%)	1.18 (0.82, 1.74)	1.22(0.84, 1.88)	1.23 (0.87, 1.79)	0.1315
Proportion of APACHE II score ≥ 15 in all ICU patients(%)	61.02(38.22,79.22)	61.89(40.52,79.69)	59.38(36.86,76.52))	0.3361
3 h SSC bundles compliance rate(%)	83.18 (61.29, 100)	86.71 (65.96, 100)	89.22 (70.00, 100) ^**†**^	0.0102
6 h SSC bundles compliance rate(%)	64.93(33.55,93.06)	74.00(39.62,96.91)	76.19 (46.88,96.67) ^**†**^	0.0031
Microbiology detection before antibiotics(%)	83.91(49.75,97.87)	84.14(60.46,97.24)	90.00 (69.62, 100) ^*†^	< 0.0001
DVT prophylaxis rate(%)	74.19(33.47,96.16)	71.70(38.05,96.28)	83.27 (47.36,97.77) ^*†^	0.0093
Unplanned endotracheal extubation rate(%)	1.10(0.14, 3.35)	1.27(0.24, 3.23)	1.29(0.34, 3.35)	0.1075
Reintubation rate within 48 h(%)	1.64(0.48, 4.44)	1.60(0.55, 3.81)	1.81(0.66, 3.79)	0.4621
Rate of unplanned transfer to ICU(%)	2.70(0.68, 8.46)	2.47(0.70, 7.77)	2.59(0.83, 7.73)	0.7667
ICU re-admission rate within 48 h(%)	0.97(0.40, 2.09)	0.89(0.32, 2.01)	1.10(0.46, 2.09)	0.1904
VAP incidence rate(%)/1000 ventilator days	11.06 (4.23, 22.70)	10.20 (4.25, 23.94)	8.05(3.13, 17.37) ^*†^	0.0002
CRBSI incidence rate(%)/1000 line days	1.30(0, 3.40)	1.39(0, 3.39)	1.24(0, 3.13)	0.6586
CAUTI incidence rate(%)/1000 line days	2.12(0.53, 4.52)	1.76(0.61, 4.41)	1.84(0.70, 4.10)	0.6703
ICU Mortality rate(%)	8.49(4.42, 14.82)	8.95(4.89, 15.70)	9.05(5.12, 15.80)	0.1075

*SSC* surviving sepsis campaign, *DVT* deep vein thrombosis, *VAP* ventilator-associated pneumonia, *CRBSI* catheter-related bloodstream infection, *CAUTI* catheter-associated urinary tract infections

^*^vs. 2015, *P* < 0.05;

^**†**^vs. 2016, *P* < 0.05;

Moreover, the 3-h and 6-h SSC bundle compliance rates in the second year of the QI program were also significantly higher than the baseline rates (3-h SSC bundle compliance rate (%): 83.18 (61.29, 100) vs. 89.22 (70.00, 100), *P* = 0.0102; 6-h SSC bundle compliance rate (%): 64.93 (33.55, 93.06) vs. 76.19 (46.88, 96.67), *P* = 0.0031). The changing trend of the proportion of septic shock patients admitted to the ICU was not significant over the 3 years (proportion of septic shock patients admitted to the ICU (%): 5.67(2.48, 12.15) in 2016, 6.34 (2.76, 13.71) in 2017, 6.67 (2.82, 14.03) in 2018, *P* = 0.0683).

### Subgroup analysis of secondary and tertiary hospitals

A total of 228 of the 586 hospitals were secondary hospitals, and 358 of the 586 hospitals were tertiary hospitals. A significantly decreased microbiology detection rate before antibiotic use, DVT prophylaxis rate, and proportion of ICU patients with APACHE II scores ≥ 15 and an increased CRBSI incidence rate was found at baseline (2016) in secondary hospitals compared with tertiary hospitals. Moreover, the tertiary hospitals had significantly higher ICU mortality rates than the secondary hospitals. In the subgroup analysis, there was a continuous decrease in the VAP incidence rate in tertiary hospitals but not in secondary hospitals during the implementation of the QI program. The comparisons of the 15 quality control indicators for secondary and tertiary hospitals are shown in Table 4.

**Table 4:** Comparison of 15 quality control indicators between second-level and tertiary hospital

Indicators	2016	2017	2018
Proportion of ICU in total inpatients(%)			
Second-level hospital	1.73 (1.19, 2.55)	1.78(1.25, 2.66) ^¶^	1.71(1.19, 2.49)
Tertiary hospital	1.55(1.10, 2.35)	1.56(1.08, 2.29)	1.58(1.09, 2.37)
Proportion of ICU in total inpatient bed occupancy(%)			
Second-level hospital	1.33 (0.96, 1.98)	1.37(0.94, 1.93)	1.28(0.92, 1.84)
Tertiary hospital	1.12(0.71, 1.53)	1.16(0.79, 1.77)	1.19(0.84, 1.74)
Proportion of APACEH II score ≥ 15 in all ICU patients(%)			
Second-level hospital	52.70 (29.94, 74.55) ^¶^	58.65 (35.75, 79.93)	55.56 (29.85, 75.38)
Tertiary hospital	65.07 (42.86, 80.04)	64.49 (46.53, 79.31)	61.12 (41.93, 76.60)
3 h SSC bundles compliance rate(%)			
Second-level hospital	75.00 (57.14, 100)	85.54 (57.74, 100)	86.75 (55.09, 100)
Tertiary hospital	84.92 (62.50, 100)	87.64 (67.46, 100)	89.83(75.00, 100) ^*^
6 h SSC bundles compliance rate(%)			
Second-level hospital	54.34 (27.27, 90.00)	68.75 (34.15, 100)	72.54 (33.19, 100)
Tertiary hospital	70.47 (39.87, 93.44)	76.27 (41.86, 94.44)	78.47 (54.55, 94.74)
Microbiology detection before antibiotics(%)			
Second-level hospital	73.76 (40.00, 95.90) ^¶^	72.12 (39.30, 96.34) ^¶^	87.40 (58.50, 99.93) ^*†^
Tertiary hospital	87.44 (62.42, 98.43)	88.49 (69.03, 97.48)	90.48 (73.77, 100) ^*^
DVT prophylaxis rate(%)			
Second-level hospital	63.33 (25.00, 96.18) ^¶^	67.12 (29.42, 95.92)	80.74 (35.87, 97.44)
Tertiary hospital	76.95 (41.67, 96.07)	74.16 (42.48, 96.30)	84.43 (51.87, 98.26)
Unplanned endotracheal extubation rate(%)			
Second-level hospital	1.55 (0, 4.76)	1.65(0, 4.17)	2.07(0.45, 4.65)
Tertiary hospital	0.93(0.29, 2.42)	1.06(0.24, 2.50)	1.06(0.33, 2.78)
Reintubation rate within 48 h(%)			
Second-level hospital	1.92 (0, 5.77)	1.62(0.19, 4.32)	2.00(0.36, 4.44)
Tertiary hospital	1.57(0.65, 3.76)	1.60(0.61, 3.27)	1.72(0.74, 3.50)
Rate of unplanned transfer to ICU(%)			
Second-level hospital	2.24 (0.45, 6.74)	2.40(0.66, 6.82)	2.69(0.76, 8.83)
Tertiary hospital	2.99(0.76, 10.05)	2.53(0.70, 8.76)	2.54(0.84, 7.28)
ICU readmission rate within 48 h(%)			
Second-level hospital	1.01 (0, 2.42)	0.85(0.22, 2.08)	1.26(0.38, 2.27)
Tertiary hospital	0.97(0.50, 1.90)	0.91(0.37, 1.98)	1.02(0.48, 1.89)
VAP incidence rate(%)			
Second-level hospital	12.75 (3.68, 31.15)	15.48 (2.79, 31.81)	9.22(2.43, 22.73)
Tertiary hospital	10.30 (4.58, 19.67)	9.04(4.59, 18.62)	7.62(3.30, 15.07) ^*†^
CRBSI incidence rate(%)			
Second-level hospital	0.47 (0, 2.47)	0.76(0, 2.84)	0.86(0, 3.17)
Tertiary hospital	1.82(0.52, 3.55)	1.68(0.53, 3.49)	1.40(0.49, 3.12)
CAUTI incidence rate(%)			
Second-level hospital	2.42 (0, 6.11)	1.42(0.19, 5.10)	1.97(0.62, 4.98)
Tertiary hospital	2.02(0.68, 4.00)	1.79(0.79, 4.21)	1.79(0.76, 3.71)
ICU Mortality rate(%)			
Second-level hospital	7.36(3.78,13.25) ¶	7.72(4.43,13.7) ¶	8.41(4.59,13.96) ¶
Tertiary hospital	9.24(4.62,16.13)	10.09(5.17,17.32)	9.66(5.52,16.42)

*SSC* surviving sepsis campaign, *DVT* deep vein thrombosis, *VAP* ventilator-associated pneumonia, *CRBSI* catheter-related bloodstream infection, *CAUTI* catheter-associated urinary tract infections

^*^vs.2015, *P* < 0.05;

^**†**^vs.2016, *P* < 0.05;

^¶^Second-level hospital vs. tertiary hospital

### Subgroup analysis based on the provincial GDP per capita

**Fig. 2:**
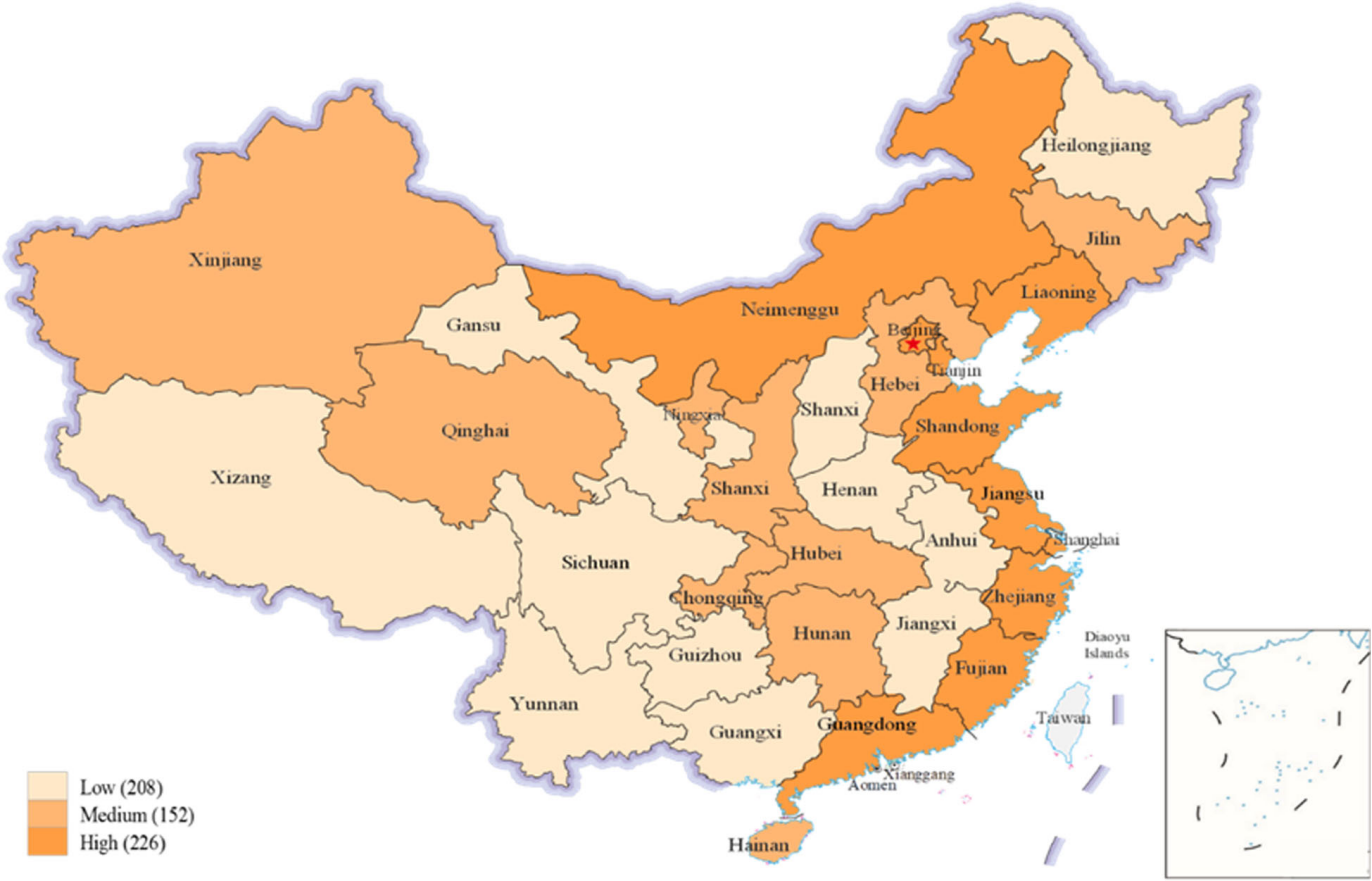
The distribution of hospital numbers based on GDP per capita in 30 provinces

Ten provinces with 226 hospitals were considered high income (GDP per capita from 107,960 to 64,168 RMB), 10 provinces with 152 hospitals were considered middle income (GDP per capita from 52,321 to 40,036 RMB), and 10 provinces with 208 hospitals were considered low income (GDP per capita from 39,462 to 26,165 RMB). The distribution of hospital number based on GDP per capita is shown in Fig. 2. The hospitals in high-income provinces had a significantly lower nurse-to-bed ratio than those in low- and middle-income provinces in 2018 (nurse-to-bed ratio: high income vs. middle income vs. low income: 2.00 (1.67, 2.50) vs. 1.83 (1.49, 2.29) vs. 1.88 (1.48, 2.38), *P* < 0.05). In the low-income provinces, the numbers of admitted hospital patients, ICU patients, and ICU beds were significantly higher in 2018 than in 2016. The structural indicators of hospitals in provinces with different levels of income are shown in Additional file 5.

In the subgroup analysis, a continuous decrease in the VAP incidence rate and a continuous increase in the microbiology detection rate before antibiotic use were found in hospitals from high-income provinces but not in hospitals from low- and middle-income provinces. (Table 5).

**Table 5:** Comparison of quality control indicators in low-, middle-, and high-income provinces

Indicators	2016	2017	2018
Proportion of ICU in total in-patients (%)			
Low GDP province	1.77 (1.25, 2.53)	1.66(1.14, 2.59)	1.72(1.15, 2.51)
Middle GDP province	1.54(1.15, 2.56)	1.60(1.2, 2.68)	1.57(1.16, 2.56)
High GDP province	1.56(1.06, 2.26)	1.60(1.08, 2.21)	1.55(1.09, 2.26)
Proportion of ICU in total inpatient bed occupancy(%)			
Low GDP province	1.12 (0.74, 1.58)	1.16 (0.84, 1.76)	1.15(0.77, 1.8)
Middle GDP province	1.08 (0.72, 1.65)	1.13 (0.73, 1.65)	1.19(0.87, 1.63)
High GDP province	1.27 (0.90, 1.87)	1.35 (0.91, 1.97) ^§^	1.36(0.94, 1.85)
Proportion of APACHE II score ≥ 15 in all ICU patients(%)			
Low GDP province	66.00 (40.07, 81.89)	62.81 (41.87, 80.25)	61.79 (39.94, 76.6)
Middle GDP province	56.88 (34.12, 75.33)	62.50 (42.31, 80.36)	60.15 (35.01, 77.39)
High GDP province	57.34 (37.44, 79.47)	60.31 (39.57, 75.17)	57.74 (36.55, 74.96)
3 h SSC bundles compliance rate(%)			
Low GDP province	85.00 (61.22, 100)	88.61 (66.67, 100)	90.28 (70.42, 100)
Middle GDP province	77.78 (60.00, 94.12)	82.43 (58.82, 100)	84.03 (67.33, 100)
High GDP province	83.33 (62.41, 100)	89.47 (66.67, 100)	89.83 (71.76, 100)
6 h SSC bundles compliance rate			
Low GDP province	66.67 (33.33, 95.16)	72.84 (41.86, 96.49)	77.14 (50.00, 95.77)
Middle GDP province	65.15 (29.31, 87.18)	67.86 (31.98, 90.12)	71.01 (40.92, 93.05)
High GDP province	64.04 (38.10, 93.33)	77.78 (43.90, 100)	78.93 (50.00, 99.09)
Microbiology detection rate before antibiotics(%)			
Low GDP province	80.24 (46.69, 99.02)	83.73 (60.81, 97.24)	89.15 (70.02, 100) ^*^
Middle GDP province	85.90 (46.96, 97.32)	84.50 (64.51, 97.36)	87.95 (69.17, 98.09)
High GDP province	85.71 (57.42, 98.33)	82.78 (57.01, 97.09)	91.34 (70.72, 100) ^*†^
DVT prophylaxis rate(%)			
Low GDP province	74.22 (30.74, 95.39)	65.40 (32.78, 94.99)	82.47 (47.02, 96.09)
Middle GDP province	74.39 (32.48, 98.42)	72.25 (33.00, 96.28)	76.80 (40.89, 96.39)
High GDP province	72.93 (41.96, 95.58)	75.46 (42.48, 97.68)	85.77 (55.62, 99.66)
Unplanned endotracheal extubation rate(%)			
Low GDP province	1.20 (0.11, 3.37)	1.32 (0.34, 3.33)	1.19 (0.36, 3.19)
Middle GDP province	1.44 (0.29, 4.17)	1.29 (0.22, 4.10)	1.41 (0.41, 3.50)
High GDP province	0.96 (0.05, 3.00)	1.23 (0.19, 2.78)	1.34 (0.30, 3.79) ^*^
Reintubation rate within 48 h(%)			
Low GDP province	1.49 (0.44, 4.00)	1.59 (0.64, 3.91)	1.61 (0.56, 3.62)
Middle GDP province	1.78 (0.43, 4.58)	1.67 (0.56, 3.45)	2.18 (0.73, 4.37)
High GDP province	1.63 (0.59, 4.44)	1.56 (0.44, 3.97)	1.87 (0.66, 3.67) ^§^
Rate of unplanned transfer to ICU(%)			
Low GDP province	2.57 (0.50, 7.21)	2.27 (0.68, 7.53)	2.49 (0.87, 8.71)
Middle GDP province	3.30 (0.74, 14.21)	2.89 (0.68, 10.16)	2.21 (0.62, 7.78)
High GDP province	2.67 (0.76, 7.59)	2.62 (0.70, 6.93)	2.76 (0.93, 7.34)
ICU readmission rate within 48 h(%)			
Low GDP province	0.97 (0.38, 2.00)	0.93 (0.34, 1.97)	1.11 (0.48, 2.16)
Middle GDP province	1.13 (0.36, 2.49)	0.84 (0.26, 2.00)	1.34 (0.62, 2.38) ^†^
High GDP province	0.93 (0.42, 2.08)	0.89 (0.37, 2.09)	1.00 (0.38, 1.72)
VAP incidence rate(%)			
Low GDP province	12.39 (4.86, 28.81)	11.64 (4.28, 25.04)	9.52 (3.27, 18.32)
Middle GDP province	9.93 (3.71, 22.87)	9.13 (3.96, 20.91)	7.83 (3.13, 15.15)
High GDP province	11.15 (4.13, 20.64)	9.77 (4.32, 23.83)	7.52 (3.02, 16.79) ^*†^
CRBSI incidence rate(%)			
Low GDP province	1.39 (0, 3.76)	1.21 (0, 3.01)	1.19 (0, 3.29)
Middle GDP province	0.98 (0, 3.25)	1.39 (0, 3.58)	1.24 (0.11, 2.98)
High GDP province	1.46 (0, 3.16)	1.60 (0, 3.62)	1.34 (0, 3.19)
CAUTI incidence rate(%)			
Low GDP province Middle GDP province	1.97 (0.47, 4.31) 2.13 (0.89, 4.97)	1.40 (0.55, 3.48) 2.02 (0.83, 4.94)	1.45 (0.59, 4.05) 2.14 (0.92, 4.59)
High GDP province	2.17 (0.59, 4.42)	1.93 (0.58, 4.55)	1.92 (0.68, 3.78)
ICU Mortality rate(%)			
Low GDP province	8.16 (4.60, 13.50)	8.39 (4.57, 16.05)	8.54 (4.65, 14.31)
Middle GDP province	7.68 (4.39, 13.12)	8.32 (4.91, 13.29)	8.90 (5.27, 14.89)
High GDP province	9.83 (4.31, 17.06)	10.15 (5.21, 15.81)	10.15 (5.67, 17.15)

*SSC* surviving sepsis campaign, *DVT* deep vein thrombosis, *VAP* ventilator-associated pneumonia, *CRBSI* catheter-related bloodstream infection, *CAUTI* catheter-associated urinary tract infections

^*^vs. 2016, *P* < 0.05

^**†**^vs.2017, *P* < 0.05

^**¶**^ vs. low income

^**§**^vs. middle income

## Discussion

We found that the implementation of a 3-year QI program period significantly decreased VAP incidence and improved DVT prophylaxis, SSC bundle compliance, and the microbiology detection rate before antibiotic use in Chinese ICUs. These findings support the hypothesis that the national QI program is effective at improving performance in the ICU setting.

Critical care medicine has made great progress, and a national quality control system for ICUs was established in China [12]. Professional qualifications for ICU attending doctors were implemented by the National Ministry of Health in China in 2009. Moreover, the specialist training project for critical care medicine started in 2019. Our present data show that ICUs have improved their attention to medical outcomes in the hospital during the past 3 years. In the present study, we focused on the effect of the QI program on three dimensions of ICU quality control indicators, including structure, processes, and outcomes, which are widely used to assess ICU performance [13, 14].

In the present study, the number of ICU beds maintained continuous growth from 2016 to 2018 in the 586 studied hospitals. Chinese society is becoming an aging society in the twenty-first century, and the number of hospitals and hospital volumes is gradually increasing. We think an aging society could also induce an increase in ICU admissions and ICU demand. However, the numbers of ICU doctors and nurses did not correspondingly increase with the increase in patients admitted to the ICU. Human resource indicators of quality control were not direct interventional targets in this study. Human resources may have been insufficient to keep pace with the increase in the number of ICU beds during the study period. Adequate human resource allocation is one of the core elements needed to ensure the quality of medical care [15]. An international investigation found that Asian ICUs had considerable variation in critical care structure, organization, and delivery, and single rooms and negative-pressure rooms were lacking [16]. Estenssoro et al. found that the availability of supporting specialists and key procedures was inadequate in Latin America [17]. Moreover, Sakr el found that a high nurse:patient ratio (> 1:1.5) was independently associated with a reduced risk of in-hospital mortality [18]. Recent literature on ICU structure in different regions/countries is summarized in Additional file 6. The annual ICU mortality rate and nurse-to-bed ratio in the present study were similar to those in US ICUs (ICU mortality, China vs USA 9% vs 10%; nurse-to-bed ratio, China vs USA 2:1 vs 1.7:1) [19]. Latin American ICUs had a lower nurse-to-patient ratio than Asian ICUs and a higher annual ICU mortality rate than US ICUs [16, 17]. Moreover, the bedside doctor-to-patient and nurse-to-patient ratios were not investigated in the present study. Here, using the doctor-to-bed ratio and nurse-to-bed ratio might roughly calculate the doctor-to-patient and nurse-to-patient ratios. In the present study, 1 doctor to 0.6 beds represented 1 doctor to 3–4 patients (equal staff 24/7). Moreover, 2 nurses to 1 bed represented 1 nurse to 2 patients (two shifts and absences for various reasons), which is similar to the results of the international investigation of Asian ICUs [16]. Here, we acknowledge that the lack of ICU human resources might impair the effectiveness of the QI program. The present study suggests that the relationship between medical human resources and ICU overexpansion was mismatched over the past 3 years. Increasing investments in ICU human resources could further improve the quality of ICU medical care in China.

For the quality control process of the QI program, we focused on sepsis bundle compliance, microbiology detection before antibiotics, and DVT prophylaxis. Our present study showed that QI program interventions could be effective at improving these process indicators. Previous studies have reported that QI programs/projects were effective at changing clinical practice [5–7]. There is a high sepsis-related mortality rate in China [20]. The 3- and 6-h SSC bundles clearly outline the clinical path for the resuscitation of patients with sepsis and have been widely accepted and promoted over the past 10 years. Rhodes revealed a 40% reduction in the odds of sepsis patients dying in the hospital when the ICU was in compliance with the 3-h bundle [21]. Leisman et al. revealed that 3-h SSC bundle compliance was associated with improved survival and cost savings [22]. However, our study did not find that increased SSC bundle compliance decreased ICU mortality. We inferred that other non-sepsis-related mortality could confound the effect of SSC compliance on sepsis-related mortality. Further studies are required to elucidate the independent effect of sepsis compliance on mortality in septic patients in China.

For the outcome indicators of the QI program, we focused on hospital-acquired infection and ICU mortality. The hospital-acquired infection has become a major issue in ICU quality control. VAP, CRBSIs, and CAUTIs have always been considered quality control indicators for ICU performance, and serial bundles were validated to reduce the incidence of acquired infections in the ICU [23–27]. In the present study, the QI program decreased the VAP incidence rate but not the CRBSI and CAUTI rates. Septic shock patients receiving mechanical ventilation therapy have a high risk of VAP. The proportion of admitted septic shock patients was 6.67% (2.82, 14.03)% in 2018. Here, we think that the improvements in sepsis bundle compliance and microbiological sampling before antibiotics might underlie the decreased VAP incidence rate.

Several potential explanations were considered for the lack of an effect on ICU mortality and other outcome indicators. First, the ICU human resource indicators were poor during the QI program period. Low doctor-to-bed and nurse-to-bed ratios were observed in 2018. Previous studies reported the effects of ICU structure indicators on ICU-acquired infections and clinical outcomes [3–5]. Second, the follow-up period was long, and the clinical setting might have changed considerably during that time. Third, it is possible that the process care indicators have very modest or negligible effects on mortality. Although all the care process indicators included in the QI program in this study were recommended by ICU experts, most have uncertain effects on mortality. For example, DVT prophylaxis may decrease adverse events but have no proven effect on mortality [28]. A multicenter controlled pre-post trial found that a multicomponent intervention involving venous thromboembolism prophylaxis changed clinical practices but not clinical and economic outcomes [29]. Moreover, several clinical studies found that early goal-directed resuscitation did not decrease the mortality of septic shock patients [30–32].

The subgroup analysis showed that the tertiary hospitals had better rates of microbiology detection before antibiotics and unplanned endotracheal extubation than secondary hospitals. Overall, in China, the medical quality of ICUs in tertiary hospitals is significantly better than that in secondary hospitals. This is related to the higher qualifications of medical staff, a higher number of patients, more financial support from the government or other sources, more standardized diagnoses and treatment processes and stricter quality management in tertiary hospitals than in secondary hospitals. In the present study, both tertiary and secondary hospitals were improved by the QI program. However, the VAP incidence rate significantly decreased in secondary hospitals. More attention should be paid to VAP in secondary hospitals. Moreover, the relationship between ICU volume and the outcomes of ICU patients is controversial. Kluge et al. found that ICU level was not associated with in-hospital and long-term mortality of ICU patients in the Netherlands [33]. Walkey et al. reported that academic hospitals in the USA with higher severe sepsis case volumes have lower severe sepsis hospital mortality without higher costs [34]. In the present study, tertiary hospitals had a higher ICU mortality rate than secondary hospitals. It is common that ICU patients with poor response to treatment will be transferred to tertiary hospitals from secondary hospitals in China. Hence, it is easy to understand a higher mortality rate in the higher ICU level. Further studies are needed to investigate the adjusted ICU mortality based on ICU level in China.

In this QI program, the impact of the regional GDP was considered in the evaluation of the effect of the QI program on ICU performance. A continuous decrease in the VAP incidence rate and a simultaneous continuous increase in the microbiology detection rate before antibiotic use were found in hospitals in high-income provinces but not in hospitals in low- and middle-income provinces. Bonell A [35] and Hurley JC [36] also observed low VAP incidence rates in high-income areas. This may be related to the financial advantages, human resource advantages and policy advantages of high-income areas in the field of health care. In the present study, the expansion of hospital and ICU volumes were obvious in low-income provinces over the 3 years of the QI program, but the corresponding ICU human resources were lower than those in high-income provinces. More ICU resources should be directed to low-income provinces to meet the increased demands on the ICUs.

Our study needs to be interpreted within the context of its strengths. We successfully assessed the effects of multifaceted QI interventions implemented as part of the national QI program led by the China-NCCQC. The trial was large, and the enrolled 586 hospitals represented 30/33 provinces of mainland China. Hence, the present study effectively reflected ICU performance at the national level. The intervention was effectively deployed by the administration of the China-NCCQC, and ICU quality control systems were established at the national, provincial and hospital levels. We used online administrative registries as our data sources and conducted a controlled pre-post trial as a practical means of evaluating the healthcare system interventions. This study design was efficient and allowed us to focus our resources on the interventions. Furthermore, we included a large number of hospitals in the study.

Several limitations should be acknowledged. First, some data on hospital mortality or 30-day outcomes, which could further indicate the effects of the interventions, were not collected in the present study. Second, compliance with some quality control processes was lacking. Third, there may have been confounding factors because of a lack of randomization and the absence of a control group. The outside factors, such as a broad range of sepsis bundles and guidelines and education on sepsis management, could drive secular trends in the improvement of sepsis care. In the present study, the objective conditions of the enrolled hospitals were comparable and stable, the interventions were clear, and the data quality was strictly controlled. Hence, this limitation might not impact the conclusion. Fourth, the corresponding outcome indicators of improvements in DVT prophylaxis and sepsis bundle compliance, such as the DVT incidence rate, pulmonary embolism incidence rate, and sepsis mortality rate, were not collected in this study. Moreover, subgroup information, such as surgical vs non-surgical patients and type of admission, was unavailable in the present study. Since our intervention consisted of a series of composite actions, it was difficult to evaluate the contribution of any single intervention in relation to the achieved results. Fifth, the same team was involved, leading to a risk of bias with regard to obtaining objective data. Moreover, the Hawthorne effect, which is a form of reaction whereby subjects improve or modify an aspect of their behavior when being studied, may also have existed in our study [5].

## Conclusions

Among critically ill patients treated in ICUs in China, the implementation of a 3-year QI program period decreased the outcome indicator of VAP incidence and improved the process care indicators of DVT prophylaxis, SSC bundle compliance and microbiology detection rate before antibiotic use. However, the QI program did not decrease ICU mortality or other outcome indicators. Moreover, medical human resources might not have kept pace with the overexpansion of ICUs in China during the past 3 years.

## Supplementary information

**Additional file 1 :** Table S1 Interventions of QI program.

**Additional file 2.** Data quality control protocols of the QI program.

**Additional file 3.** : Figure S1.Change of the total No. of admitted patients in hospital(A) and ICU(B), the number of ICU beds(C), and doctor-to-bed ratio in ICU (D) from 2016 to 2018.

**Additional file 4.** : Figure S2.Change of VAP incidence rate(A), microbiology detection rate before antibiotic use(B), DVT prophylaxis rate(C) and ICU mortality rate(D) from 2016 to 2018.

**Additional file 5.** : Table S3 Comparison of hospitals and ICU organizational characteristics in low-, middle- and high-income province.

**Additional file 6.** : Table S4 Comparison of organizational characteristics of ICU in different countries/regions.

## Data Availability

N/A

## Abbreviations

CAUTI: Catheter-associated urinary tract infection
CRBSI: Catheter-related bloodstream infection
DVT: Deep vein thrombosis
GDP: Gross domestic product
ICU: Intensive care unit
VAP: Ventilator-associated pneumonia
QI: Quality improvement
