# Supplementary material for: Effects of a national quality improvement program on ICUs in China: a controlled pre-post cohort study in 586 hospitals
Source: Crit Care. 2020 Mar 4;24:73. doi: 10.1186/s13054-020-2790-1 (PMC7057512; doi:10.1186/s13054-020-2790-1)
Supplement: Supplementary file 1 — Additional file 1 : Table S1 Interventions of QI program. [file 13054_2020_2790_MOESM1_ESM.docx]

**Effects of a national quality improvement program on ICUs in China: a controlled pre-post cohort study in 586 hospitals**

**Table S1. Interventions of QI program**

| **Items** | **Description** |
| --- | --- |
| Build local quality control team in each ICU | - Each ICU is required to build an ICU quality control team - Each ICU is required to appoint a local ICU doctor as special contact, who is responsible for submitting and reporting local data on the internet. A monthly analysis of the change of quality control indicators must be performed and reported to the whole ICU staff (doctors and nurses) - Monthly local ICU quality control conference: self-examination and correction of quality control issues, discussion of ICU acquired infections (such as VAP, CRBSI, etc.) and adverse events (such as unplanned endotracheal extubations, etc.) - Plan-Do-Check-Action (PDCA) cycle strategies to solve problems related to quality outcome indicators (such as analyzing the reasons for catheter-related blood stream infections, and proposing a local practice) |
| Training | - The special contact of each ICU receives training on interpreting 15 quality control indicators from the China-NCCQC - The special contact of each ICU is responsible for sharing study materials from the China-NCCQC by WeChat/email - Monthly local ICU quality control conference: all the staff must study the quality control material - Update recent guidelines on ICU quality controls by the China-NCCQC and province quality centers - Training materials: definition of the 15 quality control indicators, construction and management of critical care medicine, guidelines for ventilator-associated pneumonia (VAP), catheter-related blood stream infections (CRBSIs), deep vein thromboses (DVTs), catheter-associated urinary tract infections (CAUTIs), sepsis, resuscitation, PDCA cycle strategy, etc.[1,2,3,4,5,6] |
| Audits and feedback | - Annual work conference on ICU quality control at the national and province levels - China-NCCQC offers a hotline to interpret and apply the 15 quality control indicators and other issues - National center and province center randomly on-site check approximately 80 ICUs per year. Contents of the on-site check: ICU quality control team construction, PDCA for improving quality outcome indicators, training records |

1. Chinese Society of Critical Care Medicine. Guidelines for the construction and management of Intensive Care Unit (ICU) in China (2006). Zhong Guo Wei Zhong Bing Ji Jiu Yi Xue, 2006. 18(7): p. 387-388. (In Chinese).
2. Chinese Society of Critical Care Medicine. Guidelines for the diagnosis, prevention and treatment of ventilator-associated pneumonia (2013). Zhong Hua Nei Ke Za Zhi, 2013. 52(6): p. 524-543. (In Chinese).
3. Chinese Society of Critical Care Medicine. Guidelines for the prevention and treatment of intravascular catheter-related infections (2007). Zhong Hua Nei Ke Za Zhi, 2008. 28(6): p. 413-421. (In Chinese).
4. Chinese Society of Critical Care Medicine. Guidelines for severe sepsis and septic shock in China(2014). Zhong Hua Nei Ke Za Zhi,，2015,54（6）：557-581. (In Chinese).
5. Document of Guidelines for the construction and management of critical care medicine[2009],released by China’s Ministry of Health.(In Chinese).

http://www.nhc.gov.cn/wjw/gfxwj/201304/cc4ffaa8314e4ddab76788b3f7be8e71.shtml

1. Document of Medical quality control indicators for critical care medicine[2015], released by National Health Commission of the People’s Republic China (In Chinese).

http://www.nhc.gov.cn/yzygj/s3585/201504/5fa7461c3d044cb6a93eb6cc6eece087.shtml
