# Supplementary material for: Effects of a national quality improvement program on ICUs in China: a controlled pre-post cohort study in 586 hospitals
Source: Crit Care. 2020 Mar 4;24:73. doi: 10.1186/s13054-020-2790-1 (PMC7057512; doi:10.1186/s13054-020-2790-1)
Supplement: Supplementary file 2 — Additional file 2. Data quality control protocols of the QI program. [file 13054_2020_2790_MOESM2_ESM.docx]

**Effects of a national quality improvement program in ICUs in China: a controlled pre-post cohort study in 586 hospitals**

**Data quality control protocols of the QI program**

1. Setting illogical or erroneous data into the electronic system: The database of the National Clinical Improvement System (<https://ncisdc.medidata.cn/login.jsp>) was designed, and these related indicators were collected. From a logical aspect, illogical or erroneous data were defined within the electronic system on the internet (shown in the following table). These errors were automatically identified by the system and provided feedback to the data reporter. This was taken as the primary data quality control checkpoint in the system.

**Table S2. Definition of illogical and erroneous data in the electronic system**

| **Type of error** | **Parameters for determining erroneous data** |
| --- | --- |
| Illogical data | ICU bed occupancy is greater than the total bed occupancy in hospital and/or total bed occupancy is zero |
|  | The total number of patients admitted to the ICU is greater than the total number of patients admitted to the hospital |
|  | The total inpatient bed occupancy in the ICU is greater than the inpatient bed occupancy in the whole hospital |
|  | The total number of patients with an Apache II score ≥ 15 points is greater than the total number of patients admitted to the ICU |
|  | The number of inpatients who underwent microbiology detection before antibacterial agent treatment is greater than the total number of ICU inpatients using antibiotics for therapeutic purposes |
|  | The number of patients admitted to the ICU with a diagnosis of septic shock and completed a 3 h bundle is greater than the total number of patients admitted to the ICU with a diagnosis of septic shock |
|  | The number of patients admitted to the ICU with a diagnosis of septic shock and completed a 6 h bundle is greater than the total number of patients admitted to the ICU with a diagnosis of septic shock |
|  | The number of inpatients in the ICU with DVT prophylaxis is greater than the total number of inpatients in the ICU |
|  | The number of unplanned extubation patients is greater than the total number of extubation patients in the ICU |
|  | The number of unplanned ICU transfer patients is greater than the total number of inpatients admitted to the ICU |
|  | The total number of reintubations is zero but the number of reintubations within 48 hours is greater than zero |
|  | The number of patients returning to the ICU within 48 hours after discharge from the ICU is greater than the total number of patients discharged from the ICU |
|  | The number of cases of VAP in the ICU is greater than the sum of the days that each patient was put on invasive mechanical ventilation |
|  | The number of cases of CRBSI in the ICU is greater than the sum of the days that each patient had an indwelling intravascular catheter |
|  | The number of cases of CAUTI in the ICU is greater than the sum of the days that each patient had an indwelling catheter |
| Data entry errors | Number of deaths in the ICU is negative |
|  | Number of cases is not an integer |

2. Data collection: The ICUs of the enrolled hospitals that received the China-NCCQC notification were responsible for logging and filling in the specialist data and uploading them to the China-NCCQC after review by each hospital's Department of Medical Administration. The local hospital's Department of Medical Administration worked as the first-line supervisor of data quality control.

3. Data tracing: All the data were required to be traced from the local hospital's Department of Medical Administration.

4. Data check: All the submitted data from the enrolled hospitals were checked annually by the China-NCCQC. Moreover, the province center was required to check the local data monthly.
