# Supplementary material for: Effects of a national quality improvement program on ICUs in China: a controlled pre-post cohort study in 586 hospitals
Source: Crit Care. 2020 Mar 4;24:73. doi: 10.1186/s13054-020-2790-1 (PMC7057512; doi:10.1186/s13054-020-2790-1)
Supplement: Supplementary file 3 — Additional file 3. : Figure S1.Change of the total No. of admitted patients in hospital(A) and ICU(B), the number of ICU beds(C), and doctor-to-bed ratio in ICU (D) from 2016 to 2018. [file 13054_2020_2790_MOESM3_ESM.docx]

**Effects of** **a national quality improvement program in ICUs in China: a controlled pre-post cohort study in 586 hospitals**

Figure S1 Change of the total No. of admitted patients in hospital(A) and ICU(B), the number of ICU beds(C), and doctor-to-bed ratio in ICU (D) from 2016 to 2018

**
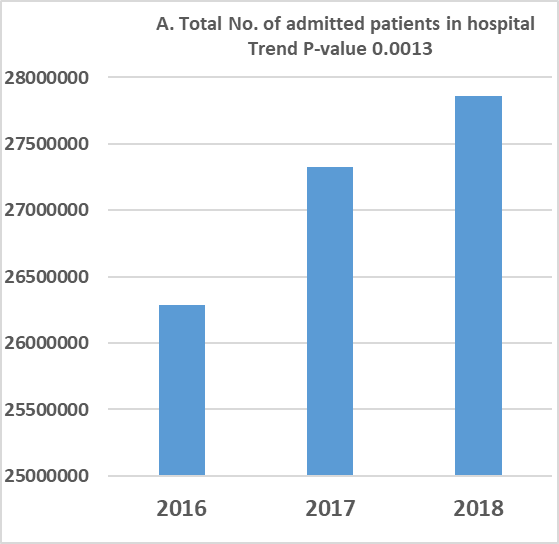

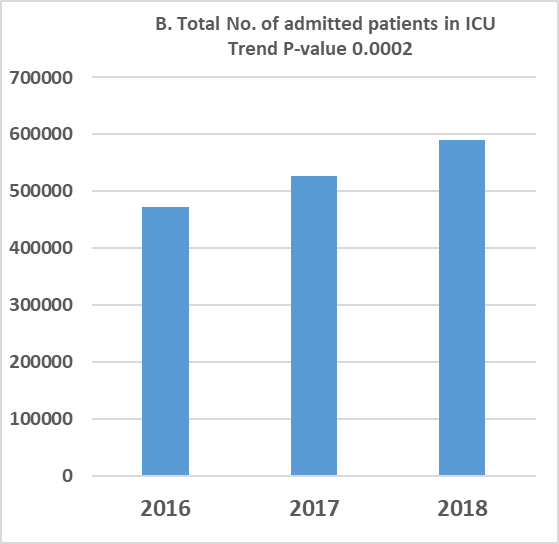
**

**
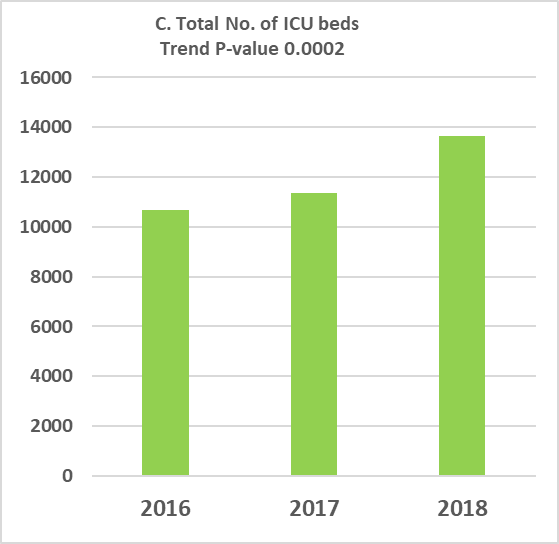

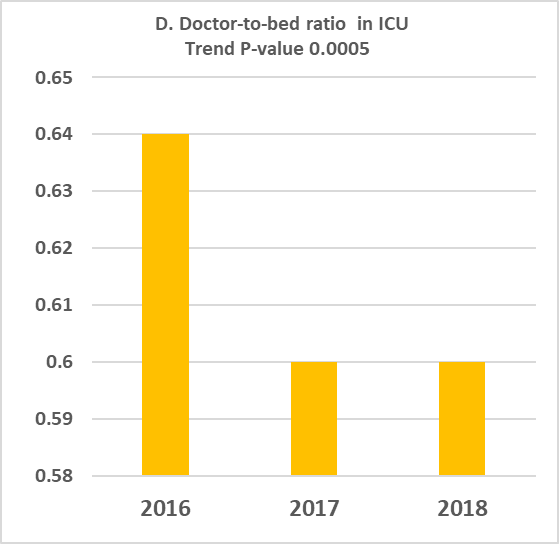
**
