# Supplementary material for: Effects of a national quality improvement program on ICUs in China: a controlled pre-post cohort study in 586 hospitals
Source: Crit Care. 2020 Mar 4;24:73. doi: 10.1186/s13054-020-2790-1 (PMC7057512; doi:10.1186/s13054-020-2790-1)
Supplement: Supplementary file 4 — Additional file 4. : Figure S2.Change of VAP incidence rate(A), microbiology detection rate before antibiotic use(B), DVT prophylaxis rate(C) and ICU mortality rate(D) from 2016 to 2018. [file 13054_2020_2790_MOESM4_ESM.docx]

**Effects of** **a national quality improvement program on ICUs in China: a controlled pre-post cohort study in 586 hospitals**

Figure S2 Change of VAP incidence rate(A), microbiology detection rate before antibiotic use(B), DVT prophylaxis rate(C) and ICU mortality rate(D) from 2016 to 2018

**
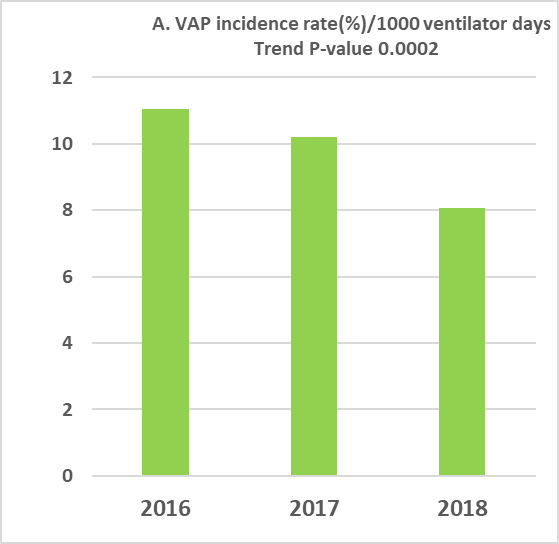

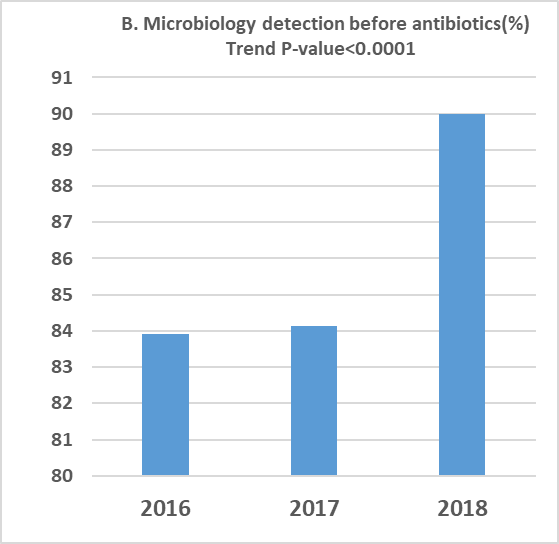
**

**
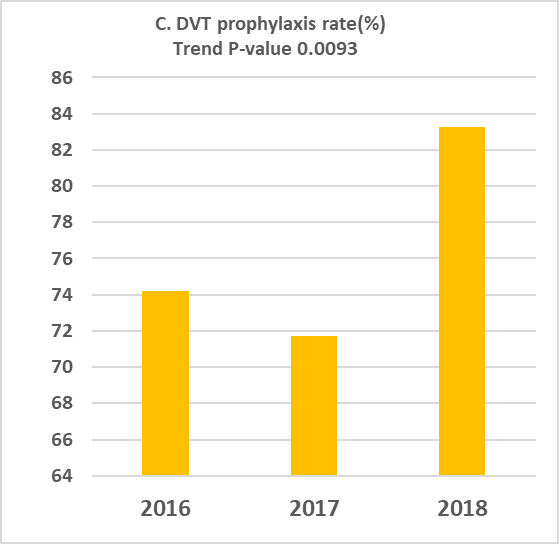

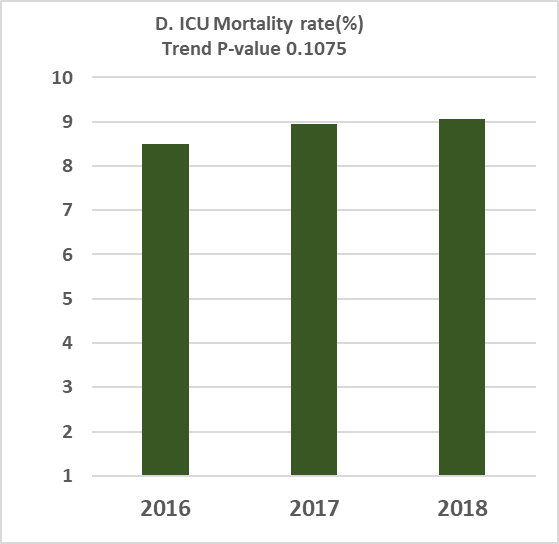
**
