# Supplementary material for: Effects of a national quality improvement program on ICUs in China: a controlled pre-post cohort study in 586 hospitals
Source: Crit Care. 2020 Mar 4;24:73. doi: 10.1186/s13054-020-2790-1 (PMC7057512; doi:10.1186/s13054-020-2790-1)
Supplement: Supplementary file 5 — Additional file 5. : Table S3 Comparison of hospitals and ICU organizational characteristics in low-, middle- and high-income province. [file 13054_2020_2790_MOESM5_ESM.docx]

**Effects of** **a national quality improvement program in ICUs in China: a controlled pre-post cohort study in 586 hospitals**

**Table S3 Comparison of hospitals and ICU organizational characteristics in low-, middle- and high-income province**

| **Indicators** | **2016** | **2017** | **2018** |
| --- | --- | --- | --- |
| Total No. of admitted patients  Low GDP province  Middle GDP province  High GDP province | 31940 (21000,46887)  29961(19161,50915)  34363 (21243,60715) | 35272 (22711,53872)  31437 (21656,50738)  36948 (21710,63708) | 38880(24479, 56942) ^*^  32028(21859, 59289)  38038(23221, 66649) |
| Total No. of admitted patients in ICU  Low GDP province  Middle GDP province  High GDP province | 500 (301, 5865)  502 (284, 867)  555(297, 843) | 575 (341, 955)  541 (345, 934)  565 (318, 893) | 662 (361, 1126) ^*^  548 (359, 999)  611 (344, 1041) |
| Total No. of hospital beds  Low GDP province  Middle GDP province  High GDP province | 1000 (600,1400)  848 (585,1347)  943 (604,1500) | 1052(636,1500)  900 (593,1390)  950 (600, 1530) | 1100 (699, 1527)  895 (607, 1479)  954 (636, 1600) |
| Total No. of ICU beds  Low GDP province  Middle GDP province  High GDP province | 13 (8, 20)  12 (9, 19)  14 (10, 21) | 13 (10, 20)  12 (9, 18)  14 (10, 21) | 15 (10, 22) ^*^  14 (10, 23)  16 (10, 26) |
| ICU: hospital bed percentage(%)  Low GDP province  Middle GDP province  High GDP province | 1.4(1.1, 1.9)  1.5(1.1, 2.0)  1.5(1.2, 2.1) | 1.4(1.1, 1.9)  1.5(1.1,1.9)  1.6(1.2, 2.0) **^¶^** | 1.6(1.1, 2.0)  1.7(1.2,2.0) **^†^**  1.7(1.3, 2.3) |
| Doctor-to-nurse ratio in ICU  Low GDP province  Middle GDP province  High GDP province | 0.31(0.25,0.38)  0.32(0.28,0.40)  0.30(0.25,0.39) | 0.30(0.24,0.38)  0.32(0.27,0.37)  0.31(0.26,0.38) | 0.30(0.24,0.38)  0.31(0.26,0.38)  0.30(0.25,0.38) |
| Doctor-to-bed ratio in ICU  Low GDP province  Middle GDP province  High GDP province | 0.60(0.47,0.83)  0.64(0.50,0.83)  0.67(0.50,0.83) | 0.60(0.46,0.75)  0.64(0.50,0.80)  0.64(0.50,0.80) | 0.55(0.42,0.75) ^*^  0.60(0.45,0.72) ^*^  0.63(0.50,0.80) |
| Nurse-to-bed ratio in ICU  Low GDP province  Middle GDP province  High GDP province | 2.00(1.54,2.50)  2.00(1.56,2.50)  2.15(1.75,2.50 ) | 2.00(1.50,2.42)  2.00(1.57,2.50)  2.14(1.77,2.50) | 1.88(1.48,2.38)  1.83(1.49,2.29)  2.00(1.67,2.50) **^¶ §^** |

GDP gross domestic product per capita.

^*^vs.2016, P<0.05;**^†^**vs.2017, P<0.05;

**^¶^**vs. low income;**^§^**vs. middle income
