# Supplementary material for: Effects of a national quality improvement program on ICUs in China: a controlled pre-post cohort study in 586 hospitals
Source: Crit Care. 2020 Mar 4;24:73. doi: 10.1186/s13054-020-2790-1 (PMC7057512; doi:10.1186/s13054-020-2790-1)
Supplement: Supplementary file 6 — Additional file 6. : Table S4 Comparison of organizational characteristics of ICU in different countries/regions. [file 13054_2020_2790_MOESM6_ESM.docx]

**Effects of** **a national quality improvement program in ICUs in China: a controlled pre-post cohort study in 586 hospitals**

**Table S4 Comparison of organizational characteristics of ICU in different countries/regions**

| **Publication**  **Year** | **Regions** | **No. of Hospitals** | **No. of hospital beds** | **No. of ICU beds/ ICU pts per year** | **Ratio of nurses to patients/beds** | **Note** |
| --- | --- | --- | --- | --- | --- | --- |
| 2016[1] | Asia (20 countries from 2013-2014) | 335 | 973.2 (mean) | 21 beds/ICU | Nurse-to-patient ratio:  1:1-1:2 in 84% ICUs  ≤1:3 in 16% ICUs | Considerable variation in critical care structure, organization, and delivery in Asia |
| 2018[2] | Latin America (9 countries: Brazil, Chile, Argentina, etc. ) | 254 | Beds< 500, 203 ICUs  Beds 500-1000, 37 ICUs  Beds>1000, 7 ICUs | Beds>20, 84 ICUs  Beds 9-20, 93 ICUs  Beds≤8, 73 ICUs | physician-to-patient  ratio was between 1:4 and 1:7 (46%)  Nurse-to-patient ratio  <1:2.1 in 177 ICUs (69%)  1:1-1:2.1 in 65 ICUs (25%)  1:1 in 3 ICUs | Annual ICU mortality was 17.8% |
| 2016[3] | International observational study ( North America, Central and South  America, Western Europe, Eastern Europe, Asia, Oceania, and  Africa: 75 counties) | 1265 | Hospital beds:  North America:  520 (460–768)  Africa:  470 (200-800)  Asia:  650 (180-100)  Eastern Europe:  563 (250-1200)  Latin America:  199 (93-309)  Oceania:  425 (300-551)  Western Europe:  550 (320-950) | ICU pts per year:  North America:  977 (684-1431)  Africa:  652 (412-1131)  Asia:  638 (400-1074)  Eastern Europe:  574 (328-1196)  Latin America:  510 (348-813)  Oceania:  944 (736-1227)  Western Europe:  695 (423-1080) | Nurse-to-patient ratio > 1:1.5 was related to in-hospital death. | 1-day prevalence of infection enrolled 13796 patients in ICU; ICU mortality was 18.2% |
| 2014[4] | United States | 69 | 611 (10-1300) | ICU pts per year  1299 (230-4556) | Ratio of beds to nurses: 1.7:1 (0.8:1–5.2:1) | Annual ICU mortality was 10.8% |

Pts, patients

**Reference**

1.Arabi YM1, Phua J, Koh Y, Du B, Faruq MO, Nishimura M, Fang WF, Gomersall C, Al Rahma HN, Tamim H, Al-Dorzi HM, Al-Hameed FM, Adhikari NK, Sadat M; Asian Critical Care Clinical Trials Group. Structure, Organization, and Delivery of Critical Care in Asian ICUs. Crit Care Med. 2016 Oct;44(10):e940-8.

2.Estenssoro E, Alegría L, Murias G, et al. Latin-American Intensive Care Network (LIVEN) Organizational Issues, Structure, and Processes of Care in 257 ICUs in Latin America: A Study From the Latin America Intensive Care Network. Crit Care Med. 2018;45(8):1325-1336.

3.Sakr Y, Moreira CL, Rhodes A, Ferguson ND, Kleinpell R, Pickkers P, Kuiper MA, Lipman J, Vincent JL; Extended Prevalence of Infection in Intensive Care Study Investigators. The impact of hospital and ICU organizational factors on outcome in critically ill patients: results from the Extended Prevalence of Infection in Intensive Care study.Crit Care Med. 2016;43(3):519-26..

4.Checkley W, Martin GS, Brown SM, et al; United States Critical Illness and Injury Trials Group Critical Illness Outcomes Study Investigators: Structure, process, and annual ICU mortality across 69 centers: United States Critical Illness and Injury Trials Group Critical Illness Outcomes Study. Crit Care Med 2014; 42:344–356
